# Patterns and associated factors of health literacy among residents aged 15–69 in Zhejiang, China: a latent profile analysis

**DOI:** 10.3389/fpubh.2025.1734757

**Published:** 2026-01-26

**Authors:** Shuiyang Xu, Yunfang Zhou, Mingyu Huang, Xinyu Wen, Xuehai Zhang, Yue Xu, Dingming Yao, Xiujing Hu, Heni Chen, Chun Chen, Xiangyang Zhang

**Affiliations:** 1 Zhejiang Provincial Center for Disease Control and Prevention, Hangzhou, Zhejiang, China; 2 School of Pharmaceutical Sciences, Wenzhou Medical University, Wenzhou, Zhejiang, China; 3 School of Medical Humanities and Management, Wenzhou Medical University, Wenzhou, Zhejiang, China; 4 Cixi Biomedical Research Institute, Wenzhou Medical University, Ningbo, Zhejiang, China; 5 The First Affiliated Hospital of Wenzhou Medical University, Wenzhou, Zhejiang, China

**Keywords:** associated factors, health literacy, LPA, patterns, Zhejiang

## Abstract

**Background:**

Health literacy plays an important role in disease prevention and control. The aim of this study is to explore the health literacy patterns and associated factors among residents in Zhejiang Province.

**Methods:**

This study included 56,863 residents aged 15–69 years from the 2024 Zhejiang Province Health Literacy Survey. Latent Profile Analysis (LPA) was used to investigate health literacy patterns, and multinomial logistic regression analysis was employed to identify associated factors. Dominance analysis was performed to compare the relative contribution of the main variables associated with health literacy.

**Results:**

The analysis identified three distinct health literacy profiles: low literacy (15.13%), moderate literacy (32.24%), and relatively high literacy (52.63%). The low literacy group was characterized by an older demographic (with an average age of 58.71 years), lower educational attainment (20.72% had no formal education), a higher proportion of farmers (52.93%), and a significant share of low-income individuals (40.98%). Multinomial logistic regression and dominance analysis revealed that education level, age, and occupation were the most important associated factors of health literacy.

**Conclusion:**

The study findings highlighted the heterogeneity in health literacy among various population groups and emphasized the need for targeted interventions. This study provides empirical evidence to inform precision health promotion strategies in developed regions of China.

## 1 Introduction

Health literacy plays an important role in disease prevention and control. As a fundamental component of overall health and well-being, health literacy enables individuals to effectively manage health behaviors, make informed health-related decisions, and achieve positive health outcomes (1). The World Health Organization (WHO) defines health literacy as “the cognitive and social skills which determine the motivation and ability of individuals to gain access to, understand and use information in ways which promote and maintain good health” (2). Studies demonstrated that low health literacy is significantly associated with multiple adverse health outcomes, particularly in chronic disease management and healthcare utilization (3). By contrast, improving health literacy can promote healthy behaviors, enhance disease self-management capabilities, and reduce healthcare burdens (4). Recognizing health literacy's pivotal role, WHO has advocated targeted global action plans to enhance it, with health literacy's promotion now a public health goal in many countries (5, 6).

In 2008, China initiated a systematic survey of health literacy (7, 8). A study indicated that from 2008 to 2022, the proportion of the Chinese population with sufficient health literacy had increased from 6.48% to 27.78%. Specifically, by 2022, more than two-thirds of the population is classified as having insufficient health literacy (9). Zhejiang, located in eastern China and recognized as a paradigm of developed provinces in the country, has a health literacy level that serves as a quintessential case study in the fields of health promotion and public health governance. A study shows that by 2022, the proportion of Zhejiang's population with sufficient health literacy had reached 33.08%, demonstrating the province's exemplary progress in this regard (10). The *Healthy Zhejiang 2030 Action Plan* clearly designates health literacy as an important indicator and calls for the establishment of a closed-loop system for monitoring and intervention (11). Despite the overall high health literacy, certain subpopulations in Zhejiang—such as older adults, rural residents, and individuals with lower educational level—remain at risk of inadequate health literacy (9). At the same time, the current health literacy policies focus on extensive coverage of the entire population, but fail to take into account the heterogeneity within the population, and lack targeted measures and refined interventions for different groups (12).

As one of China's most economically developed and populous provinces, Zhejiang is experiencing advanced stages of demographic aging and socioeconomic transformation, conditions that are increasingly shared by other regions nationwide. Therefore, examining health literacy patterns in Zhejiang may provide an informative early reference for understanding emerging challenges and guiding future health literacy interventions in other parts of China (9).

Many previous studies have predominantly focused on individuals with specific health conditions (such as cancer or hypertension) or specific age groups (such as adolescents or older adults) (13, 14), which inherently limits the generalizability of research findings (15, 16). Additionally, limited studies have delved into the patterns of health literacy (17). In recent years, the field of health literacy research has increasingly emphasized the importance of adopting comprehensive and multi-dimensional assessment methods (18). Previous studies have indicated that health literacy is complex and heterogeneous, and it is a complex construct jointly influenced by multiple factors (19, 20). However, traditional methodologies often rely on total health literacy scores or average scores measured under different assessment criteria (21, 22). Although this approach facilitates statistical analysis, it may mask important heterogeneities within the population, leading to the neglect of the health literacy characteristics of specific sub-populations (15). In provinces with large and diverse populations such as Zhejiang, where overall health literacy levels are relatively high, the reliance on average-based analyses is particularly likely to obscure vulnerable subgroups. To date, large-scale, population-based studies applying person-centered approaches such as the Latent Profile Analysis (LPA) to examine health literacy heterogeneity in Zhejiang remain scarce, limiting the evidence base for targeted intervention design.

To address this limitation, this study employs the LPA (19, 20). The LPA method is based on individuals' performance characteristics in multiple health literacy dimensions and objectively identifies potential heterogeneous subpopulations by constructing probabilistic models (20, 23). We explore the latent profiles of health literacy among residents aged 15–69 in Zhejiang province, analyse the patterns of health literacy, and examine the characteristic differences and associated factors across distinct latent profiles, with the findings aiming to provide a scientific basis for formulating targeted health intervention strategies (16, 24, 25).

## 2 Materials and methods

### 2.1 Data sources and sample

The data of this study were derived from the 2*024 Zhejiang Province Health Literacy Survey*, which is a large-scale population-based assessment project. The target population of the survey covered non-collective resident population with Chinese nationality aged 15–69 years old in all counties (cities and districts) of Zhejiang Province, excluding residents living in military bases, hospitals, prisons, nursing homes and dormitories. The formula for estimating the minimum sample size of each stratum in the surveillance of residents' health literacy and tobacco use prevalence carried out in all counties (cities and districts) is as follows:


$$N=\frac{\mu_{\alpha }^{2}\times p\left(1-p\right)}{\delta^{2}}\times deff$$

Based on the residents' health literacy level of 38.36% in Zhejiang Province in 2022, *p* was set as 0.3836. With an allowable relative error of 15%, the corresponding absolute error was 0.05754. Using μ_α_ = 1.96 and *deff* = 1, the minimum required sample size for each stratum was calculated to be 274 participants. Sampling was conducted in accordance with national guidelines using a stratified multistage probability proportional to size (PPS) approach. Specifically, four townships were selected in each city, county, and district, followed by the selection of two communities (villages) within each township. From each community (village), 100 households were randomly sampled, and one participant per household was selected using the Kish grid method for face-to-face interviews. Each community (village) required at least 80 completed questionnaires. Ultimately, a total of 56,863 valid questionnaires were collected through household surveys conducted by professionally trained interviewers, who gathered multidimensional information from residents, including health literacy assessments.

The inclusion criteria of this study were permanent residency in Zhejiang and age between 15 and 69 years. All data collectors underwent standardized professional training to ensure consistency and reliability in data collection. All collected data were complete, and no participants were excluded due to missing or invalid data. A total of 56,863 samples were included in the final analysis.

### 2.2 Health literacy

The measurement of health literacy adopts the Chinese Health Literacy Scale. The scale contains 50 questions, covering six health literacy dimensions necessary for solving practical health problems. It includes six core dimensions: (1) scientific view of health (eight items; score range 0–11), (2) infectious disease literacy (six items; score range 0–7), (3) chronic disease literacy (nine items; score range 0–12), (4) safety and first-aid literacy (10 items; score range 0–14), (5) medical care literacy (11 items; score range 0–14), and (6) health information literacy (six items; score range 0–8). Responses to true or false and single-choice questions were assigned a score of 0 or 1, and responses to multiple-choice questions were assigned a score of 0 or 2 (26).

### 2.3 Predictive factors

By reviewing previous studies (27, 28), we included following information such as age, sex (female/male), residence (urban/rural), local household registration (no/yes), marital status (married/single, divorced or widowed), educational level (no formal education/primary school/middle school/college or above), occupation (student/farmer/worker/enterprise personnel/personnel of public institutions/other), household income per capita [low income (≤12,500 CNY)/lower-middle income (12,501–25,000 CNY)/upper-middle income (25,001–50,000 CNY)/high income (>50,000 CNY)], smoking status (no/yes), number of chronic diseases (0 chronic disease/1 chronic disease/≥2 chronic diseases), and self-rated health (poor/fair/good) as exposure variables in the analysis.

### 2.4 Statistical analysis

First, the six-dimensional health literacy scores were analyzed using Latent Profile Analysis (LPA), with each dimension treated as a continuous observed indicator. LPA is a model-based clustering technique grounded in finite mixture modeling, which posits that the population consists of a finite number of mutually exclusive latent profiles. In this framework, the observed indicators are assumed to be conditionally independent within each latent profile. To identify the optimal latent structure, model estimation started with a one-profile solution, and successive models with an increasing number of profiles were fitted. Model fit was then evaluated using the Akaike Information Criterion (AIC), the Bayesian Information Criterion (BIC), and entropy, with lower values of AIC and BIC indicating better model fit and higher entropy reflecting greater classification accuracy. Following the identification of latent profiles, descriptive analyses were conducted, presenting variables as means [Standard Deviation (SD)] or medians [Interquartile Range (IQR)] or frequencies with percentages. Subsequently, group comparisons for continuous variables were performed using one-way analysis of variance (one-way ANOVA), while categorical variables were compared using the chi-squared test. All tests were two-sided. To further examine associations, a multinomial logistic regression model was constructed to assess the relationships between variables and latent profiles of health literacy, with the low health literacy group specified as the reference category. Finally, dominance analysis was employed to quantify the relative contribution of predictive factors in different latent profiles (5, 18). All statistical analyses were performed using Stata version 17.0.

## 3 Results

### 3.1 Description of sample

This sample consists of 56,863 residents (mean age: 55.22 years, SD: 13.43; age range: 15–69 years), among whom 53.61% are female, 7.13% have no formal education, 30.70% are farmers, mainly with local household registration (92.81%), and the majority have an education level of a middle school (49.30%).

### 3.2 The latent profile analysis

**Figure 1:**
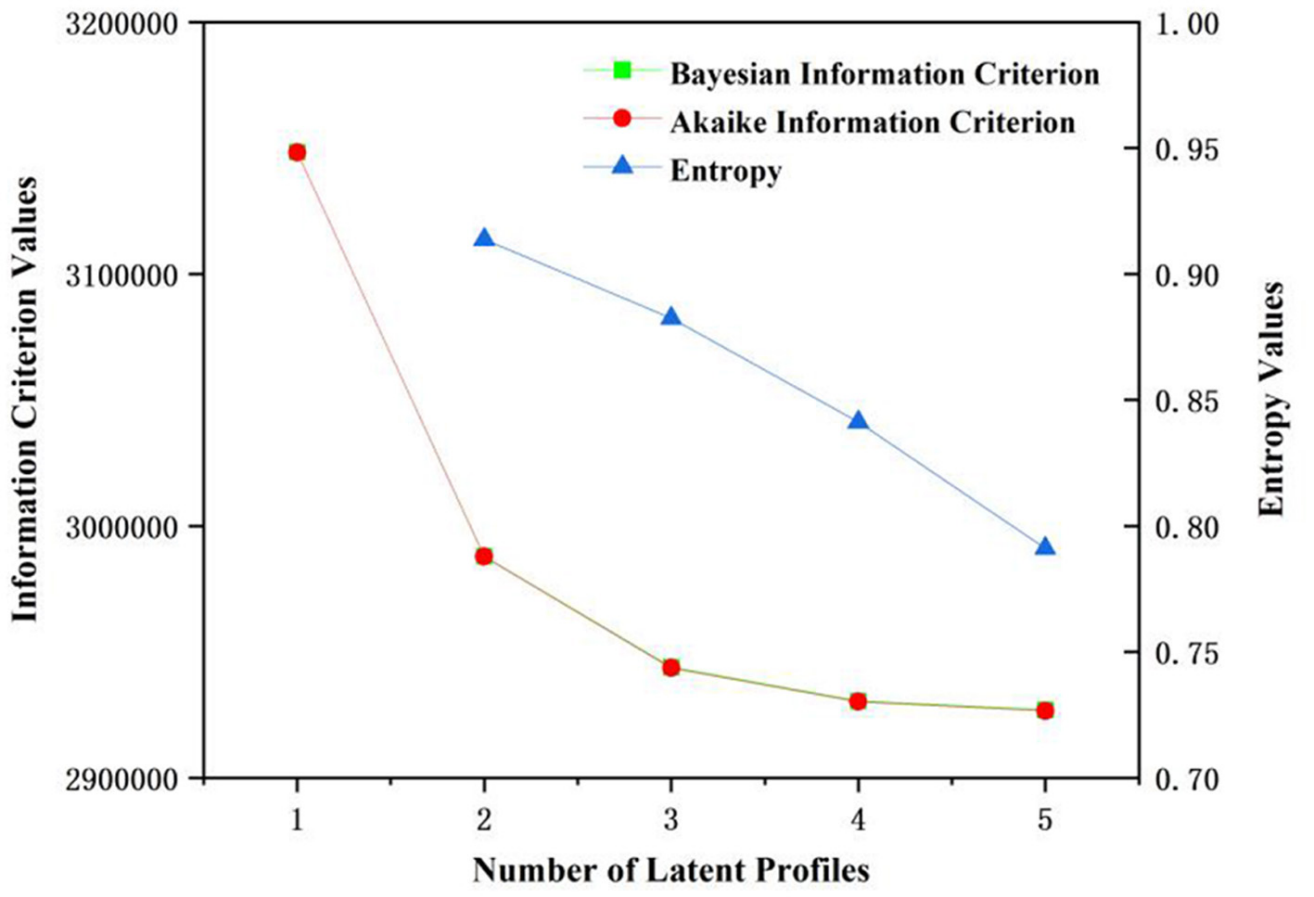
Elbow-plot of selected fit indices for latent profiles analysis.

The model fit indices for the latent profile analysis of health literacy are presented in Figure 1 and Table 1. Models with 1 to 5 potential profiles were constructed stepwise using the 6 dimensions of health literacy entries as exogenous variables. As the number of profiles increased, the AIC (decreasing from 3,148,282.4 to 2,926,535.0), BIC (decreasing from 3,148,389.8 to 2,926,893.0). Notably, the 3-profile model demonstrated a higher entropy value (0.88254562) compared to the 4-profile model (0.84127824). Based on these results, the 3-profile model was identified as the optimal fit for the data, revealing three distinct latent profiles of health literacy.

**Table 1:** Model fit in dices for latent profile analysis of health literacy.

**Model**	**LL**	**BIC**	**AIC**	**Entropy**
1	−1,574,129.2	3,148,389.8	3,148,282.4	–
2	−1,493,867.6	2,987,943.3	2,987,773.3	0.91373538
3	−1,471,799.2	2,943,883.1	2,943,650.4	0.88254562
4	−1,465,069.7	2,930,500.7	2,930,205.4	0.84127824
5	−1,463,227.5	2,926,893.0	2,926,535.0	0.79105189

LL, log-likelihood; BIC, Bayesian information criterion; AIC, akaike information criterion.

**Figure 2:**
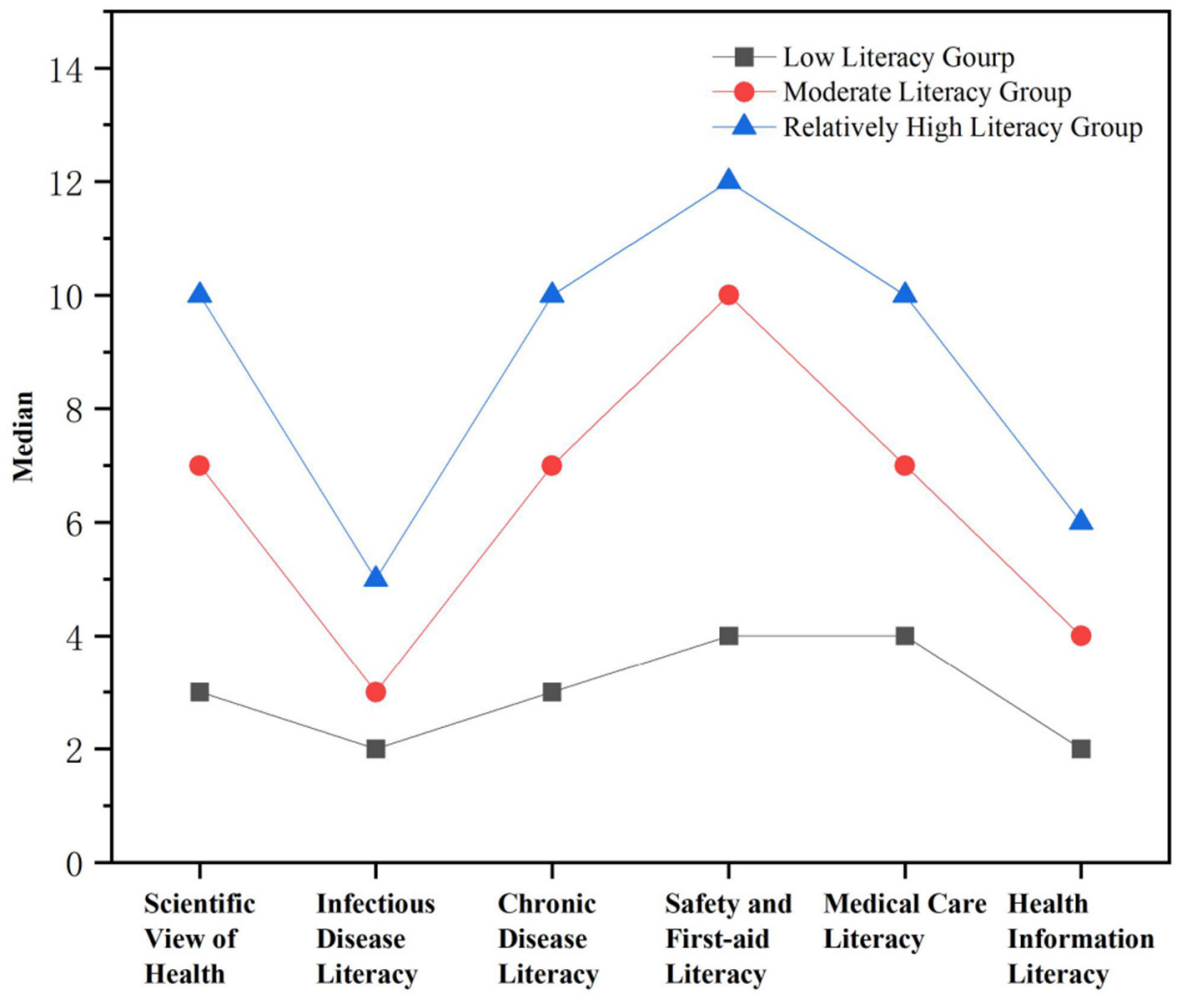
Median Values for six dimensions of the three-profile model of health literacy.

Based on the scoring results across the six dimensions of the health literacy scale, participants were categorized into three groups: low literacy group, moderate literacy group, and relatively high literacy group. As shown in Figure 2, latent profile analysis of health literacy revealed a clear three-profile hierarchical structure: (1) low literacy group (*n* = 8,602, 15.13%): exhibited the lowest scores across all dimensions, indicating deficient basic health knowledge. (2) Moderate literacy group (*n* = 18,335, 32.24%): had intermediate but uneven performance, suggesting partial skill mastery. (3) High literacy group (*n* = 29,926, 52.63%): demonstrated comprehensive advantages, with scores approaching full marks.

### 3.3 Characteristics of participants across different latent profile memberships

The descriptive data for participants in the three latent profiles is shown in Table 2. Significant disparities were noted among the three groups concerning associated factors.

**Table 2:** Comparison between three groups.

**Variables**	**Total *n* = 56,863**	**Low literacy group** ***n* = 8,602**	**Moderate literacy group *n* = 18,335**	**Relatively high literacy group *n* = 29,926**	***p***
**Age**	50.11 ± 13.43	58.71 ± 8.75	54.88 ± 11.30	44.72 ± 13.35	**<0.001** ^**a**^
**Sex**					
Female	30,487 (53.61)	4,850 (56.38)	9,828 (53.60)	15,809 (52.83)	<0.001^b^
Male	26,376 (46.39)	3,752 (43.62)	8,507 (46.40)	14,117 (47.17)	
**Residence**					
Urban	23,366 (41.09)	3,123 (36.31)	7,160 (39.05)	13,083 (43.72)	<0.001^b^
Rural	33,497 (58.91)	5,479 (63.69)	11,175 (60.95)	16,843 (56.28)	
**Local household registration**					
No	4,087 (7.19)	487 (5.66)	1,155 (6.30)	2,445 (8.17)	<0.001^b^
Yes	52,776 (92.81)	8,115 (94.34)	17,180 (93.70)	27,481 (91.83)	
**Marital status**					
Married	46,249 (81.33)	7,269 (84.50)	15,570 (84.92)	23,410 (78.23)	<0.001^b^
Single, divorced or widowed	10,614 (18.67)	1,333 (15.50)	2,765 (15.08)	6,516 (21.77)	
**Educational level**					
No formal education	4,054 (7.13)	1,782 (20.72)	1,865 (10.17)	407 (1.36)	<0.001^b^
Primary school	11,116 (19.55)	3,384 (39.34)	5,214 (28.44)	2,518 (8.41)	
Middle school	28,036 (49.30)	3,295 (38.30)	9,925 (54.13)	14,816 (49.51)	
College or above	13,657 (24.02)	141 (1.64)	1,331 (7.26)	12,185 (40.72)	
**Occupation**					
Student	1,701 (2.99)	34 (0.40)	256 (1.40)	1,411 (4.71)	<0.001^b^
Farmer	17,458 (30.70)	4,553 (52.93)	7,366 (40.17)	5,539 (18.51)	
Worker	9,858 (17.34)	1,740 (20.23)	3,807 (20.76)	4,311 (14.41)	
Enterprise personnel	12,480 (21.95)	807 (9.38)	2,772 (15.12)	8,901 (29.74)	
Personnel of public institutions	6,751 (11.87)	185 (2.15)	994 (5.42)	5,572 (18.62)	
Other	8,615 (15.15)	1,283 (14.91)	3,140 (17.13)	4,192 (14.01)	
**Household income per capita (CNY)**					
Low income (≤12,500)	14,656 (25.77)	3,525 (40.98)	5,575 (30.41)	5,556 (18.57)	<0.001^b^
Lower-middle (12,501–25,000)	14,999 (26.38)	2,441 (28.38)	5,439 (29.66)	7,119 (23.79)	
Upper-middle (25,001–50,000)	17,373 (30.55)	2,070 (24.06)	5,364 (29.26)	9,939 (33.21)	
High income (>50,000)	9,835 (17.30)	566 (6.58)	1,957 (10.67)	7,312 (24.43)	
**Smoking status**					
No	46,670 (82.07)	6,869 (79.85)	14,682 (80.08)	25,119 (83.94)	<0.001^b^
Yes	10,193 (17.93)	1,733 (20.15)	3,653 (19.92)	4,807 (16.06)	
**Number of chronic diseases**					
0	41,340 (72.70)	5,167 (60.07)	12,004 (65.47)	24,169 (80.76)	<0.001^b^
1	12,382 (21.78)	2,638 (30.67)	4,985 (27.19)	4,759 (15.91)	
≥2	3,141 (5.52)	797 (9.26)	1,346 (7.34)	998 (3.33)	
**Self-rated health**					
Poor	2,285 (4.02)	646 (7.51)	947 (5.16)	692 (2.31)	<0.001^b^
Fair	16,683 (29.34)	3,138 (36.48)	6,084 (33.18)	7,461 (24.93)	
Good	37,895 (66.64)	4,818 (56.01)	11,304 (61.66)	21,773 (72.76)	

Continuous variables are presented as mean ± SD, and categorical variables are presented as frequency (percentage).

a *p* value calculated by one-way ANOVA,

b *p* value calculated by chi-squared test.

Middle school: junior high school/High school/secondary/vocational high school.

Household income per capita (CNY): household per capita income was calculated as total annual household income divided by household size. Income groups were defined based on quartiles of the sample distribution.

There were significant differences among different health literacy groups. The low literacy group had a significantly higher mean age (58.71 years) than the moderate (54.88 years) and high (44.72 years) literacy groups. Females constituted a higher proportion (56.38%) in the low literacy group compared to the moderate and high literacy groups, with rural residents accounting for 63.69%. In terms of educational level, the proportions of participants with no formal education (20.72%) and primary school education (39.34%) in the low literacy group were significantly higher than those in the other two groups. Regarding occupational distribution, farmers comprised 52.93% of the low literacy group. Moreover, the proportions of low-income individuals (40.98%), chronic disease prevalence (30.67% with one disease, 9.27% with two or more diseases), smoking rate (20.15%), and poor self-rated health status (7.51%) were all significantly higher than those in the moderate and high literacy groups.

### 3.4 Multinomial logistic regression analysis

Table 3 presents the results of the multinomial logistic regression analysis.

**Table 3:** Multinomial logistic regression of different latent profiles of health literacy.

**Variables**	**Moderate literacy group vs. low literacy group**		**Relatively high literacy group vs. low literacy group**	
	**OR (95% CI)**	***p***	**OR (95% CI)**	***p***
**Age**	0.978 (0.975–0.982)	**<0.001**	0.944 (0.941–0.948)	**<0.001**
**Sex (Ref: Female)**				
Male	1.034 (0.970–1.103)	0.305	1.066 (0.997–1.140)	0.060
**Residence (Ref: Urban)**				
Rural	1.003 (0.949–1.061)	0.905	1.154 (1.088–1.225)	**<0.001**
**Local household registration (Ref: No)**				
Yes	1.392 (1.238–1.566)	**<0.001**	2.082 (1.846–2.349)	**<0.001**
**Marital status (Ref: Single, divorced or widowed)**				
Married	1.148 (1.065–1.237)	**<0.001**	1.427 (1.316–1.549)	**<0.001**
**Educational level (Ref: No formal education)**				
Primary school	1.315 (1.212–1.426)	**<0.001**	2.452 (2.169–2.771)	**<0.001**
Middle school	2.158 (1.979–2.354)	**<0.001**	8.912 (7.883–10.075)	**<0.001**
College or above	4.552 (3.684–5.624)	**<0.001**	64.657 (51.789–80.723)	**<0.001**
**Occupation (Ref: Student)**				
Farmer	0.745 (0.504–1.101)	0.140	0.732 (0.501–1.070)	0.107
Worker	0.749 (0.507–1.105)	0.145	0.685 (0.469–0.998)	0.049
Other enterprise personnel	0.847 (0.573–1.252)	0.405	0.966 (0.662–1.410)	0.857
Personnel of public institutions	1.02 (0.673–1.547)	0.924	1.372 (0.917–2.051)	0.124
Other	0.878 (0.593–1.297)	0.513	0.844 (0.577–1.232)	0.379
**Household income per capita (CNY) (Ref: Low income (** ≤ **12,500))**				
Lower-middle (12,501–25,000)	1.271 (1.190–1.357)	**<0.001**	1.468 (1.367–1.577)	**<0.001**
Upper-middle (25,001–50000)	1.311 (1.221–1.406)	**<0.001**	1.643 (1.525–1.771)	**<0.001**
High income (>50,000)	1.377 (1.234–1.538)	**<0.001**	2.029 (1.816–2.266)	**<0.001**
**Smoking status (Ref: No)**				
Yes	0.901 (0.833–0.974)	0.009	0.774 (0.713–0.839)	**<0.001**
**Number of chronic diseases (Ref: 0)**				
1	1.059 (0.996–1.127)	0.067	1.065 (0.996–1.138)	0.066
≥2	1.052 (0.952–1.163)	0.319	1.032 (0.921–1.156)	0.591
**Self–rated health (Ref: Poor)**				
Fair	1.074 (0.960–1.202)	0.211	1.173 (1.027–1.339)	0.018
Good	1.089 (0.974–1.218)	0.134	1.251 (1.098–1.427)	0.001

Statistical significance was defined as *p* < 0.001. OR, odds ratio. Bold values indicate statistical significance (*p* < 0.001).

Compared with the low literacy group, the relatively high literacy group was more likely to exhibit the following characteristics: being married [OR = 1.434, 95% CI (1.320–1.558)], higher educational attainment [college or above: OR = 62.391, 95% CI (49.711–78.304)], higher income levels [upper-middle income: OR = 1.546, 95% CI (1.436–1.664); high income: OR = 1.877, 95% CI (1.681–2.095)], urban residence [OR = 1.145, 95% CI (1.079–1.215)], and non-smoking status [OR = 0.760, 95% CI (0.700–0.826)].

The moderate literacy group was more likely to exhibit the following characteristics: being married [OR = 1.155, 95% CI (1.070–1.247)], higher educational attainment [middle school: OR = 2.173, 95% CI (1.990–2.373); college or above: OR = 4.572, 95% CI (3.681–5.680)], higher income levels [upper-middle income: OR = 1.267, 95% CI (1.182–1.359); high income: OR = 1.322, 95% CI (1.185–1.475)], and lower smoking probability [OR = 0.879, 95% CI (0.812–0.951)].

### 3.5 Relative importance between variables

Table 4 presents the results from the dominance analysis.

**Table 4:** Standardized dominance weights of independent variables based on dominance analysis.

**Variables**	**Standardized Dominance Weights**	**Ranking**
Education level	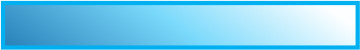 44.9%	1
Age	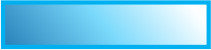 25.9%	2
Occupation	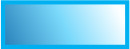 15.7%	3
Household income per capita (CNY)	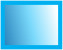 6.7%	4
Number of chronic diseases	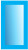 2.8%	5
Self-rated health	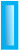 1.7%	6
Marital status	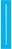 0.8%	7
Local household registration	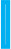 0.8%	8
Smoking status	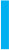 0.4%	9
Residence	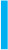 0.3%	10
Sex	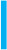 0.2%	11

The results show that educational level is the most significant associated factor, accounting for the highest proportion of 44.9% (Ranking 1). The analysis also showed the importance of age, which accounted for 25.9% (Ranking 2). Furthermore, occupation is also an important factor, which accounted for 15.7% (Ranking 3).

## 4 Discussion

This study applied the LPA to investigate the health literacy patterns among residents aged 15–69 years in Zhejiang Province, China. Three distinct latent profiles were identified: low (15.13%), moderate (32.24%), and relatively high (52.63%) literacy groups. By capturing population heterogeneity, this person-centered approach provides an empirical basis for the development of targeted and differentiated health literacy interventions. Importantly, following the LPA-based classification, educational level, age, and occupation consistently emerged as the most influential factors associated with health literacy, underscoring the necessity of prioritizing these factors in intervention strategies.

The contrasting sociodemographic and health-related characteristics observed between the low and relatively high literacy groups reflect underlying structural inequalities rather than isolated individual factors. Individuals in the low literacy group were more likely to occupy socially disadvantaged positions characterized by older age, rural residence, lower educational level, and poorer health status, which tend to cluster and reinforce one another across the life course (29, 30). From a person-centered perspective, these co-occurring disadvantages converge to form a distinct health literacy profile characterized by limited access to health resources, reduced cognitive and informational capacity, and greater exposure to health risks, including chronic diseases and smoking (4, 18, 31). In contrast, the relatively high literacy group represents an accumulation of social and health advantages, including higher education, urban residency, and better health, which collectively facilitate the acquisition and application of health-related information (32, 33). This pattern highlights the cumulative and interactive nature of social factors in shaping health literacy profiles.

Educational level, age, and occupation emerged as key associated factors of health literacy, a finding that is consistent with existing literature. Educational level was strongly associated with health literacy, with individuals holding a college degree or above demonstrating significantly higher levels of health literacy. Previous studies in Europe and other settings have similarly reported lower health literacy among individuals with lower educational levels (34, 35). Education facilitates health-related decision-making by enhancing information-processing capacity and scientific literacy, such as the ability to understand medical terminology (20, 34, 36).

Age is the second most significant associated factor, with a significantly higher proportion of elderly individuals in the low literacy group. This association may reflect age-related cognitive decline affecting memory and comprehension, as well as the digital divide that limits older adults' access to health information through modern communication technologies (37, 38). Consistent with these findings, international studies have shown a negative correlation between age and health literacy, underscoring age as a key social determinant (34, 39).

Occupation further contributed to disparities in health literacy, with farmers accounting for a substantially higher proportion of individuals in the low literacy group. This pattern likely reflects the combined effects of lower income, high labor intensity, and relatively limited educational opportunities, which together constrain the ability to obtain, understand, and apply health information (40).

The three factors—educational level, age and occupation—jointly constitute the core factors influencing health literacy, with their combined weight reaching as high as 86.5%; this high proportion indicates that any effective intervention strategy can anchor the core resources to these three factors. Factors such as income (6.7%), number of chronic diseases (2.8%), self-rated health (1.7%), marital status (0.8%), and local household registration (0.8%) are moderately associated with residents' health literacy levels, but their influence is relatively limited. The factors with the weakest impact are smoking status (0.4%), place of residence (0.3%), and sex (0.2%).

In terms of research implications, the findings highlight the need for longitudinal tracking systems and cross-regional comparative studies to better understand the dynamic relationships between social factors and health literacy and to assess the generalisability of health literacy profiles across different socioeconomic contexts. In terms of health intervention practices, the results suggest that targeted strategies focusing on key groups defined by educational level, age, and occupation may be more effective than uniform population-wide approaches. At the policy level, establishing coordinated mechanisms across education, elderly care, and employment sectors, while prioritizing health literacy as a policy objective and promoting inter-departmental data sharing, may support sustained improvements in population health literacy.

Several limitations of this study should be acknowledged. First, the cross-sectional design limits the ability to draw causal inferences regarding the relationships between associated factors and health literacy profiles. Second, while this study provides empirical evidence on health literacy profiles and their associated factors, the findings are primarily intended to inform understanding at the analytical level. The translation of these results into specific policy measures or intervention strategies requires further context-specific research to assess feasibility and effectiveness.

## Data Availability

The data used and analysed during the current study are available from the corresponding author on reasonable request.
